# The Pathogenesis and Medical Treatment of Depression: Opportunity and Challenge

**DOI:** 10.3390/neurolint17080120

**Published:** 2025-08-04

**Authors:** Mengjiao Xu, Zhiyu Zhang, Zhoudong Zhang, Dong Liu, Yanguo Shang, Chenglun Tang, Weipeng Wang, Huanqiu Li, Bengang You, Hanjie Ying, Tao Shen

**Affiliations:** 1 College of Biotechnology and Pharmaceutical Engineering, Nanjing Tech University, Nanjing 211816, China; 201961109010@njtech.edu.cn (M.X.); 202261218263@njtech.edu.cn (Z.Z.); liudong@njtech.edu.cn (D.L.); syg2023@njtech.edu.cn (Y.S.); yinghanjie@njtech.edu.cn (H.Y.); 2 College of Pharmaceutical Sciences, Soochow University, Suzhou 215006, China; 20244026008@stu.suda.edu.cn (Z.Z.); wangweipeng@suda.edu.cn (W.W.); 3 Jiangsu Institute of Industrial Biotechnology, JITRI Co., Ltd., Nanjing 210000, China; tangchenglun308@163.com

**Keywords:** depression, serotonin, HPA, inflammatory factors, gut–brain axis, therapeutic drugs

## Abstract

Depression is a common mental disorder with high economic burden, characterized by high disability and mortality rates. The etiology of depression remains unclear to date, and there are various hypotheses regarding the pathogenesis of depression in clinical practice, including the monoamine neurotransmitter hypothesis, the hypothalamic–pituitary–adrenal (HPA) axis dysregulation hypothesis, the inflammatory cytokine hypothesis, and the neurotrophic factor hypothesis. These theories offer specific directional aid in the clinical management of individuals suffering from depression. Medicinal intervention stands as a critical approach within the spectrum of depression treatments, and this article reviews the specific mechanisms of different hypotheses on the pathogenesis of depression in recent years, as well as the research progress on related therapeutic drugs.

## 1. Introduction

Depression is characterized by persistent sadness, sleep/appetite disturbances, anhedonia, and social anxiety. Severe cases involve delusions or suicidal ideation [1]. Post-COVID-19, global anxiety/depression prevalence rose by 27.6% [2]. Over 350 million people are affected worldwide; China’s incidence is 6%, affecting ~95 million. By 2030, depression is projected to be the leading global disease burden [3]. It increases comorbidity risk (e.g., hypertension, diabetes, CVD) and mortality [4,5], impacting public health and productivity (e.g., ~6 lost work hours/week, higher unemployment) [6,7].

Despite neuropsychiatric advances, the precise pathogenesis remains elusive [8]. Current research implicates monoaminergic dysfunction [9], HPA axis impairment [10], reduced brain derived neurotrophic factor (BDNF) [11], altered neuroplasticity [12], inflammation [13], and gut microbiome imbalance [14]. Treatment response typically takes ≥4 weeks, often accompanied by side effects (sexual dysfunction, GI issues, anxiety) [15]. Investigating mechanisms and developing novel, effective, well-tolerated antidepressants is crucial. This review analyzes depression pathogenesis hypotheses and summarizes current therapeutics. Therefore, it is essential to further investigate the underlying mechanisms of depression and explore novel medications that exhibit exceptional efficacy, excellent tolerability, minimum side reactions, and broad applicability. This review aims to provide a comprehensive analysis of the various hypotheses explaining the causes of depression. Additionally, we present a summary of the existing medications that have demonstrated therapeutic effects on depression. The objective is to offer references for the early detection, prevention, and advancement of new antidepressant treatments.

## 2. Methods of Literature Search

### 2.1. Search Strategy

A systematic literature search was conducted following the Preferred Reporting Items for Systematic Reviews and Meta-Analyses (PRISMA) guidelines. The search encompassed electronic databases including Web of Science, PubMed and Embase from 1 January 2010 to 1 June 2025. Key search terms and their combinations included: Depression pathogenesis; Monoamine hypothesis; HPA axis; Neuroinflammation; Oxidative stress; Neuroplasticity; Gut–brain axis; Antidepressants; Herbal antidepressants; Novel therapies. Boolean operators (AND/OR) were used to refine the search. Additional articles were identified through manual screening of reference lists from relevant reviews and primary studies.

Boolean Search String:

(“Depression” OR “Major Depressive Disorder” OR “Depressive Disorder”) AND (“Pathogenesis” OR “Etiology” OR “Mechanism” OR “Monoamine Hypothesis” OR “HPA axis” OR “Hypothalamic–Pituitary–Adrenal Axis” OR “Neuroinflammation” OR “Inflammatory Cytokines” OR “Oxidative Stress” OR “Neuroplasticity” OR “Neurotrophic Factors” OR “BDNF” OR “Gut–Brain Axis” OR “Gut Microbiota”) AND (“Drug Therapy” OR “Antidepressants” OR “Pharmacotherapy” OR “Monoamine Oxidase Inhibitors” OR “Tricyclic Antidepressants” OR “Selective Serotonin Reuptake Inhibitors” OR “Serotonin-Norepinephrine Reuptake Inhibitors” OR “Atypical Antidepressants” OR “Herbal Medicine” OR “Novel Therapies”) NOT (“Animals” OR “Animal Models” NOT “Humans”).

### 2.2. Inclusion and Exclusion Criteria

Inclusion criteria: Original research articles; Studies focusing on depression pathogenesis mechanisms or drug therapies; Articles published in English. Exclusion criteria: Case reports, non-peer-reviewed publications, and conference abstracts; Studies on comorbidities not directly related to depression; Articles with insufficient methodological detail or irrelevance to core themes; Articles with insufficient methodological detail or irrelevance to core themes.

### 2.3. Study Selection Process

**Figure 1:**
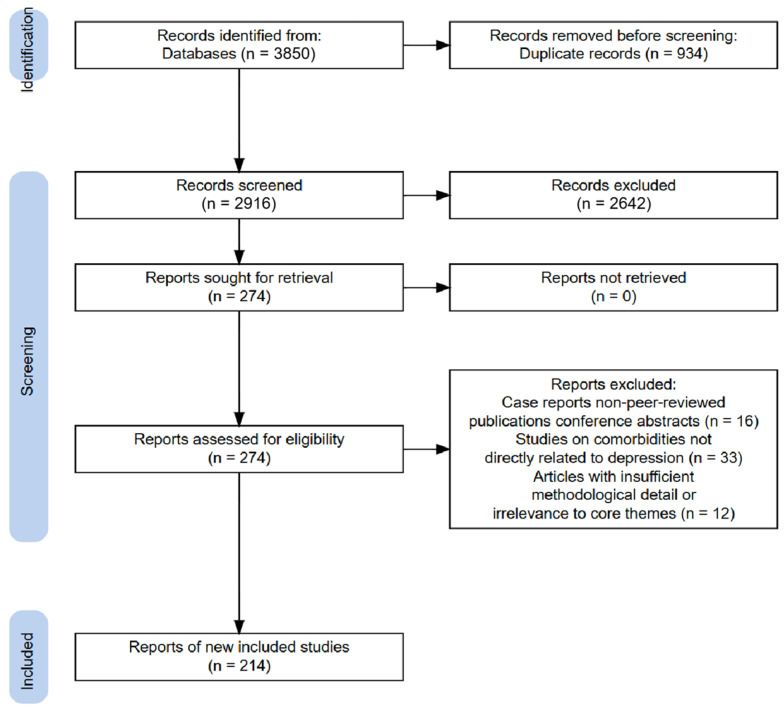
PRISMA flow diagram of the included studies (created de novo).

All identified records underwent a three-stage screening process: Initial screening: Titles and abstracts were assessed for relevance. Full-text review: Potentially eligible articles were evaluated for methodological rigor and alignment with review objectives. Final inclusion: Data from selected studies were synthesized into thematic sections (pathogenesis hypotheses, drug therapies). The screening workflow is summarized in the PRISMA flowchart below (Figure 1).

### 2.4. Data Extraction, Synthesis and Quality Assessment

Data from included studies were extracted using a standardized template, capturing: Study characteristics, pathophysiological mechanisms, treatment outcomes and risk of bias indicators (randomization, blinding, attrition). Findings were thematically organized into sections addressing pathogenesis hypotheses (Section 3) and therapeutic strategies (Section 4).

To ensure methodological rigor and minimize bias, a structured quality assessment was performed using standardized tools tailored to study design: Randomized Controlled Trials (RCTs) were evaluated using the *Cochrane Risk-of-Bias Tool, assessing randomization processes, deviations from intended interventions, missing outcome data and outcome measurement; Observational Studies underwent assessment via the Newcastle-Ottawa Scale (NOS); Mechanistic Studies were scrutinized using a “custom 9-item checklist” evaluating critical parameters including: Pathway-specific modulation approaches, Dose–response relationships, Reproducibility metrics and Appropriateness of statistical methods.

## 3. Pathogenesis of Depression

### 3.1. Hypothesis of Monoamine Neurotransmitters

**Figure 2:**
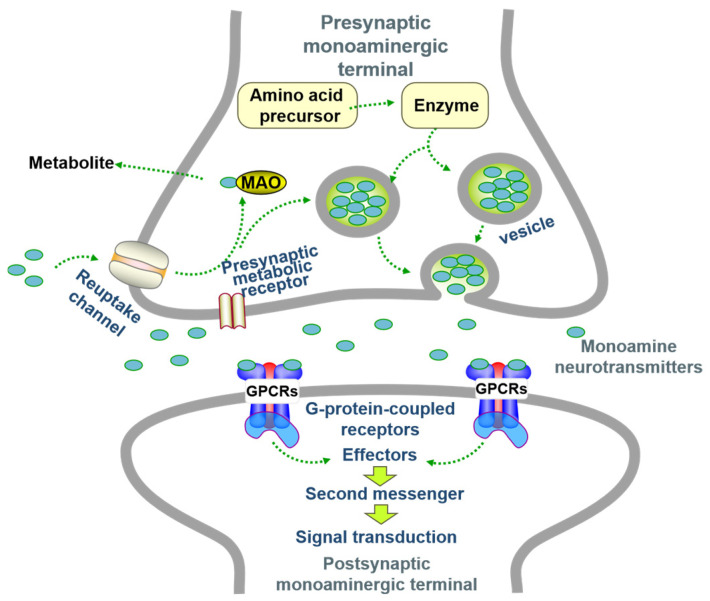
The steps of monoamine neurotransmitter transmission at the synapse (adapted from the reference [26]).

Proposed in 1972 [16], this hypothesis links depression to reduced availability/dysfunction of monoamines (Norepinephrine (NE/NA), Serotonin (5-HT), Dopamine (DA)) in the synaptic cleft, disrupting mood/cognitive signaling [17,18]. NE modulates prefrontal function (attention/behavior) [19]; 5-HT regulates pain/neuroendocrine functions; low levels cause anxiety [20]; DA affects motor/reward systems [21]. Enzymes governing monoamine synthesis/metabolism/transport are key. Monoamine oxidase (MAO) degrades monoamines, reducing transmission [22]. Stress impairs tryptophan hydroxylase (TPH) function, lowering brain 5-HT [23]. Dysfunctional neurotransmitter transporters are implicated [24,25,26,27]. Receptor abnormalities (e.g., reduced 5-HT receptors [28], presynaptic α2-adrenoceptor hypersensitivity [29]) also contribute and offer treatment targets [30] (Figure 2).

### 3.2. Impairment of the HPA Axis

**Figure 3:**
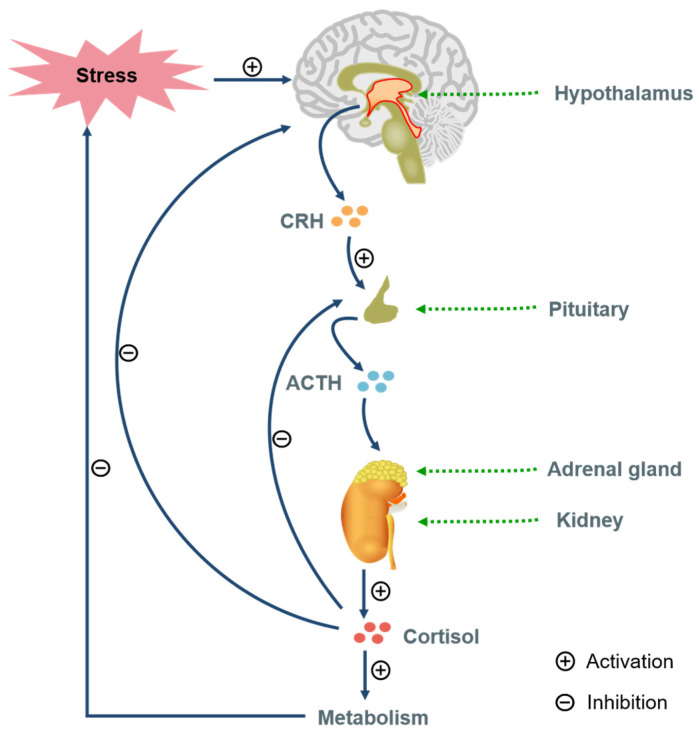
The regulation of stress response by the hypothalamic–pituitary–adrenal axis (adapted from Reference [35]).

The HPA axis regulates stress responses. Stress triggers hypothalamic corticotropin-releasing hormone (CRH) release, stimulating pituitary adrenocorticotropic hormone (ACTH), leading to adrenal glucocorticoids (GCs) secretion [31]. Chronic stress causes HPA dysfunction in 40–60% of patients, featuring hypercortisolemia, impaired GC feedback, and receptor signaling defects [32,33] (Figure 3). Depressed patients show elevated cerebrospinal fluid CRH and reduced frontal CRH receptors [34,35,36]. GCs are vital for stress response and homeostasis [37]. Chronic stress reduces GR activity, suppresses feedback, and alters behavior [28]. Elevated cortisol in depression causes neuronal degeneration (e.g., hippocampus, Prefrontal cortex) [38].

### 3.3. The Role of Inflammatory Cytokines and Oxidative Stress

**Figure 4:**
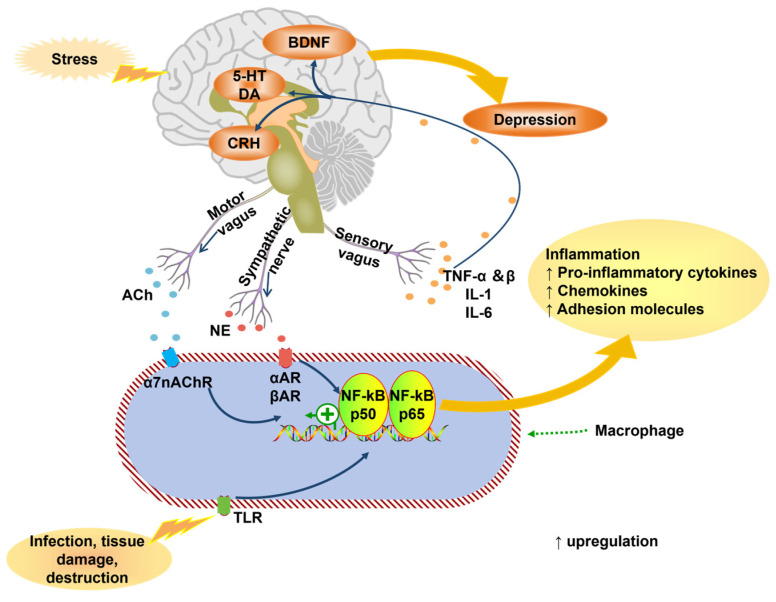
The connection between inflammatory cytokines and depression (adapted from the reference [41]). ACh, acetylcholine; TNF, tumor necrosis factor; IL, Interleukin; nAChR, nicotinic acetylcholine receptor; AR, adrenergic receptor; NF-kB, nuclear factor-kB; TLR, toll-like receptors.

Inflammation and oxidative stress are key contributors to depression pathophysiology. Pro-inflammatory cytokines (e.g., IL-6, TNF-α, IL-1β) dominate over anti-inflammatory counterparts, disrupting neuroendocrine/immune responses [39,40,41]. These cytokines activate pathways like indoleamine 2,3-dioxygenase (IDO), diverting tryptophan metabolism from serotonin synthesis toward neurotoxic kynurenine metabolites [42,43,44,45,46,47,48]. NLRP3 inflammasome activation promotes Caspase-1-mediated IL-1β/IL-18 release, exacerbating neuroinflammation [49,50,51,52,53]. TLR4 signaling (triggered by endogenous ligands like HMGB1) amplifies cytokine release via NF-κB [54,55,56,57] (Figure 4).

Oxidative stress interacts bidirectionally with inflammation. Reactive oxygen species (ROS) activate inflammatory pathways (NF-κB, MAPK) and cause mitochondrial dysfunction, impairing energy metabolism and promoting neuronal apoptosis [58,59,60]. Depressed patients show elevated oxidative markers (MDA, 8-OHdG) and reduced antioxidants (SOD, catalase, glutathione) [61,62]. This vicious cycle contributes to neuronal dysfunction and treatment resistance, suggesting antioxidants may augment antidepressant efficacy [63].

### 3.4. Hypothesis of Neuroplasticity and Neurotrophic

#### 3.4.1. Neuroplasticity Hypothesis

Neuroplasticity encompasses the brain’s structural and functional adaptability in response to experience, stress, and disease, crucial for neuronal development and morphology [64]. In depression, impaired neuroplasticity disrupts neural circuits governing emotion, cognition, and stress response [65]. Depression involves reduced hippocampal volume/neurogenesis, correlating with symptom severity [66]. Synaptic plasticity (LTP/LTD) affects neurotransmitter function [19]. Stress (via HPA axis), neuroinflammation, oxidative stress, and GABAergic dysfunction impair neuroplasticity, forming a complex basis for depression [67,68,69]. This hypothesis underscores neuroplasticity’s central role in depression pathophysiology and its potential as a therapeutic target.

#### 3.4.2. Neurotrophic Hypothesis

**Figure 5:**
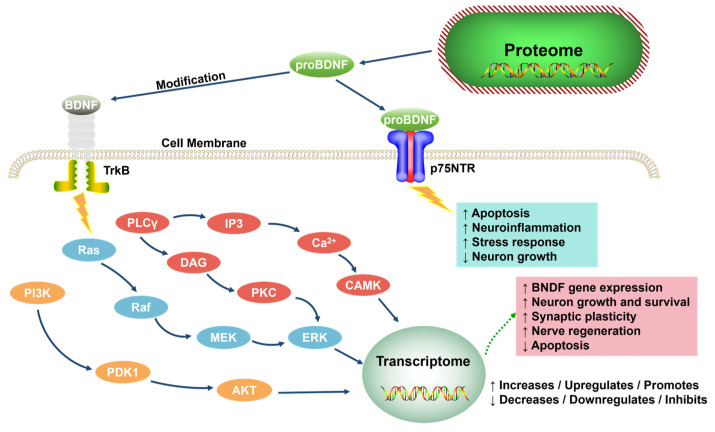
The role of neurotrophic factors in the function of the nervous system (adapted from the reference [72]). proBDNF, the precursor form of BDNF; TrkB, tropomyosin receptor kinase B; NTR, neurotrophin receptor; PLCγ, phospholipase Cγ; IP3, inositol 1,4,5-trisphosphate; DAG, diacylglycerol; PKC, protein kinase C; CAMK, calcium/calmodulin-dependent protein kinase; Ras, rat sarcoma; Raf, rapidly accelerated fibrosarcoma; MEK, MAPK/ERK kinase; ERK, extracellular signal-regulated kinase; PI3K, phosphoinositide 3-kinase; PDK1, phosphoinositide-dependent kinase 1; AKT, serine/threonine-protein kinase B.

BDNF is central, supporting neuron survival, learning, memory, and mood via TrkB receptor signaling (MAPK, PLCγ, PI3K pathways) [70,71,72] (Figure 5). proBDNF (binding p75NTR) promotes apoptosis; mBDNF (binding TrkB) enhances plasticity and is neuroprotective. Reduced BDNF impairs hippocampal neurogenesis/synaptic plasticity, mood regulation, and stress coping [73,74,75,76,77,78]. Antidepressants increase BDNF expression [79,80,81], making it a potential biomarker.

In summary, BDNF plays multiple roles in the pathogenesis of depression, including influencing neurogenesis, synaptic plasticity, neuroprotection, emotional regulation, and the ability to cope with stress. Therefore, BDNF is not only crucial for understanding the pathophysiology of depression but may also be a key target for the development of new therapeutic strategies.

### 3.5. Dysfunction of Gut Microbiota

**Figure 6:**
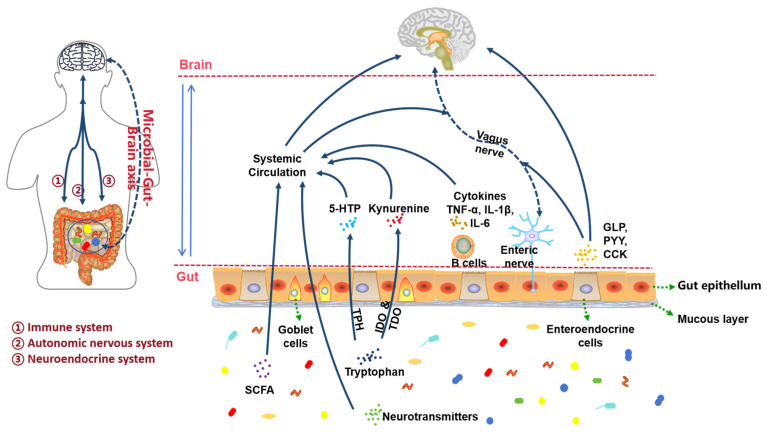
The pathways of microbiota-gut–brain axis (adapted from the reference [89]). SCFA, short chain fatty acid; TPH, tryptophan hydroxylase; IDO, indoleamine 2,3-dioxygenase; TDO, tryptophan 2,3-dioxygenase; 5-HTP, 5-hydroxytryptophan; TNF, tumor necrosis factor; IL, interleukin; GLP, glucagon-like peptide; PYY, peptide YY; CCK, cholecystokinin.

The gut microbiota, essential for health, is often dysregulated in depression, showing reduced diversity and shifts towards pro-inflammatory bacterial profiles [82,83,84,85,86]. Fecal transplants from patients induce depressive-like behaviors in rodents [87]. The microbiota-gut–brain (MGB) axis communicates via autonomic, immune, and endocrine pathways [88,89] (Figure 6).

#### 3.5.1. Autonomic Nervous System Pathway

Gut microbiota metabolites (short-chain fatty acids (SCFAs), neurotransmitters, amino acid derivatives) signal via the enteric nervous system and vagus nerve to the central nervous system (CNS) [89,90,91]. Microbiota regulate central processes including neurogenesis, neuronal activity, and glial function [92]. Microbes regulate SCFA production, enhancing colonic 5-HT synthesis via TPH1 in Enterochromaffin Cells [23,93,94]. Stress-induced Lactobacillus reduction lowers IDO inhibition, increasing kynurenine and depressive behaviors [95,96]. SCFAs modulate ENS activity and microglial maturation [97,98,99]; exogenous SCFAs improve depression-related parameters [100,101].

#### 3.5.2. Immune System Pathways

Increased gut permeability allows bacterial translocation (e.g., Lipopolysaccharide LPS), activating immune cells and microglia, causing neuroinflammation and blood–brain barrier (BBB) damage [102,103]. Probiotics (e.g., *B. infantis* CCFM687, *L. reuteri* NK33, *B. adolescentis* NK98) reverse HPA overactivation and inflammation [104,105,106]. Stress increases permeability and bacterial translocation linked to MAPK p38/Nrf2 pathways [107,108]. LPS also activates IDO, reducing 5-HT [109,110,111,112].

#### 3.5.3. Endocrine System Pathways

Stress alters microbiota, affecting HPA axis function [89,113]. Early-life/maternal stress increases cortisol and pro-inflammatory cytokines, dysregulating HPA feedback [114,115]. Depressive rats show microbiota changes and increased hypothalamic CRH, mirroring CRH injection effects [116]. Gut hormones (e.g., Peptide YY, PYY) act as signaling molecules between microbiota and brain [117,118,119].

Despite extensive research and various hypotheses proposed in recent years, the etiology and pathogenesis of depression are still not fully understood. However, researchers from different perspectives raise questions that are inevitably interconnected and complementary to each other, which will help in better understanding depression.

## 4. Drug Therapy for Depression

Treatment depends on severity. Mild cases may use psychotherapy/lifestyle changes [120]. Moderate-severe depression requires pharmacotherapy, evolving from monoamine oxidase inhibitors/tricyclic antidepressants (MAOIs/TCAs) to selective serotonin reuptake inhibitors (SSRIs), serotonin and NE reuptake inhibitors (SNRIs), and atypicals. These pharmaceutical compounds exert their effects through a variety of mechanisms, from modulating monoaminergic neurotransmission to targeting neurotrophic signaling cascades [121]. Herbal medicines show promise via multi-target actions [122,123].

### 4.1. Monoamine Oxidase Inhibitor and Tricyclic Antidepressants

In the 1950s, the first generation of antidepressants, including MAOIs and tricyclic antidepressants, emerged. These drugs work by inhibiting monoamine oxidase activity and blocking the reuptake of 5-HT and NE, respectively, thereby increasing their concentrations in the synaptic cleft, which clinically results in the improvement of depressive symptoms [124,125].

MAOIs (e.g., Phenelzine, Isocarboxazid) inhibit monoamine oxidase, increasing synaptic monoamine concentrations (e.g., 5-HT, DA) to alleviate depression [126]. They show efficacy in atypical depression and some treatment-resistant cases [127]. However, MAOIs require strict dietary tyramine avoidance due to hypertensive crisis risk and cause side effects (dizziness, insomnia) [128]. A recent study demonstrated the efficacy of MAOIs in treatment-resistant depression (TRD) unresponsive to multiple antidepressant classes, including SSRIs, SNRIs, and atypical antidepressants. Patients treated with either phenelzine or tranylcypromine for 6 months exhibited significant symptomatic improvement, manifested as enhanced mood, sleep quality, cognitive function, and social engagement. Concurrently, substantial reductions exceeding 58% were observed in both Hamilton Depression Rating Scale (HDRS) and Beck Depression Inventory (BDI) scores. Mild adverse effects—including insomnia, headaches, and orthostatic hypotension—were reported in all subjects, though these remained manageable with supportive care [129].

TCAs (e.g., Amitriptyline, Imipramine) primarily inhibit 5-HT/NE reuptake, elevating synaptic neurotransmitter levels [130]. They are cost-effective and prevent relapse but cause significant anticholinergic effects (dry mouth, constipation, blurred vision), cardiotoxicity, and cognitive impairment [124,125,131]. Tertiary amine TCAs (e.g., amitriptyline) exhibit greater toxicity than secondary amines, with overdose mortality risks (e.g., dothiepin: 53.3 deaths/million prescriptions) [132].

### 4.2. Selective Serotonin Reuptake Inhibitors

SSRIs (e.g., Fluoxetine, Sertraline) block presynaptic 5-HT reuptake, increasing synaptic serotonin and promoting neuroplasticity [133]. They are first-line for mild-moderate depression due to better safety and tolerability vs. TCAs [134]. Common limitations include sexual dysfunction (e.g., reduced libido) [135] and withdrawal symptoms (dizziness, dysesthesia) upon discontinuation. Additionally, abrupt cessation of SSRIs can result in withdrawal symptoms such as dizziness and dysesthesia. A meta-analysis of 14 studies (*n* = 4459) reported a pooled incidence of antidepressant withdrawal symptoms at 53.6% (95% CI: 43.2–63.7%), with the highest incidence for paroxetine (78.2%; 95% CI: 65.1–87.2%) and lowest for fluoxetine (43.5%; 95% CI: 31.2–56.7%) [136]. Since different patients may respond differently to various SSRIs, their use should be under the guidance of a professional doctor.

### 4.3. Serotonin-Norepinephrine Reuptake Inhibitors

SNRIs (e.g., Venlafaxine, Duloxetine) inhibit both 5-HT and NE reuptake, offering broader efficacy than SSRIs and potentially faster onset for some symptoms [137,138]. They minimally inhibit cytochrome P450 but risk serotonin syndrome with MAOIs [139]. Adverse effects include hypertension (13.1% (95% CI: 9.8–17.3%) at >300 mg/day venlafaxine), nausea (41.2%, (95% CI: 34.5–48.3%)), urinary dysfunction (10.4% (95% CI: 7.1–15.0%) with milnacipran), and hyperhidrosis [140,141]. Withdrawal syndromes occur, especially with short-half-life agents [142].

### 4.4. Atypical Antidepressants

This heterogeneous class acts via diverse mechanisms (e.g., 5-HT receptor modulation, NE/DA enhancement) distinct from conventional antidepressants [143]. Some agents (e.g., Bupropion, Agomelatine) exhibit rapid onset, fewer anticholinergic effects than TCAs, and efficacy beyond depression (e.g., anxiety, sleep improvement) [144,145]. However, they may cause sedation, weight gain, or activation [146]. Trazodone improves sleep but may increase suicide risk in insomnia [147]; agomelatine commonly causes headache (10.3% (*n* = 339, single-study data without CI)) and rhinitis (6.7%) [148]. Withdrawal reactions remain a concern [149].

### 4.5. Herbal and Emerging Agents

Traditional herbal medicines and early-stage compounds offer multi-target mechanisms but require further validation. Key examples are summarized in Table 1.

### 4.6. Novel Drug Treatment Strategies

(R,S)-Ketamine, an NMDA receptor antagonist, rapidly alleviates depressive symptoms via its metabolite (2R,6R)-hydroxynorketamine, which activates AMPA receptors [163,164]. Its S-(+)-isomer (esketamine) is approved for treatment-resistant depression (TRD) and acute suicidality [165]. AXS-05 (dextromethorphan/bupropion), targeting NMDA, showed clinical efficacy in 2023 [166]. In addition, another NMDARs also showed meaningful antidepressant activity in clinical phase III trials [167]. Ketamine’s GABA interneuron inhibition is crucial for its effects [165], and GABA_A_ targeting agents (brexanolone, zuranolone) are FDA-approved [166].

Psilocybin, a 5-HT_2A_ receptor agonist, received FDA “breakthrough therapy” designation for TRD [168]. It enhances glutamate release and promotes long-term neural plasticity in cortico-limbic circuits [172], spurring research into 5-HT_2A_ agonists [170]. Cariprazine (D_2_/D_3_ partial agonist), initially approved for schizophrenia/bipolar disorder, shows adjunctive potential for bipolar depression and TRD [171,172,173]. In addition to the above drugs, recent clinical studies have also focused on novel antidepressants, such as Ezogabine (a KCNQ channel-acting drug) [174] and LY341495 (an mGlu2/3 receptor antagonist) [175]. Exploration of highly active compounds continues to drive novel target discovery [176]. Therefore, in-depth exploration of highly active antidepressants and systematic elucidation of their mechanisms of action are key directions for advancing antidepressant drug development.

## 5. Limitations and Future Directions

### 5.1. Critical Appraisal

Despite advances in understanding depression pathogenesis, significant limitations persist in translating findings to clinical practice: (1) Heterogeneity in biomarker data (e.g., cytokines, oxidative stress markers, microbiome profiles) due to methodological variations, clinical diversity (depression subtypes, comorbidities), and demographic confounders, limits diagnostic utility; (2) Preclinical success of IDO inhibitors has not translated to human trials due to compensatory pathway activation (e.g., TDO upregulation), poor blood–brain barrier penetration, and lack of validated biomarkers for patient stratification; (3) Microbiome findings exhibit poor reproducibility across studies, influenced by geography, diet, sequencing methods, and medication confounders; human causality remains correlational despite suggestive fecal transplant data; (4) Target engagement evidence for novel agents (e.g., NLRP3 inhibitors, kynurenine modulators) is predominantly derived from animal models, with limited human mechanistic validation. Table 2 synthesizes key evidentiary gaps and quality assessments using the GRADE framework.

### 5.2. Future Priorities

Define depression endotypes via multi-omics. Develop brain-penetrant IDO inhibitors with predictive biomarkers. Establish standardized microbiome protocols. Implement longitudinal biomarker studies during treatment.

## 6. Conclusions

The pathogenesis of depression is complex, involving multiple factors such as biochemistry, environment, and psychosocial aspects. To date, it has not been possible to isolate a single pathological process that determines the development and progression of the disease. With in-depth research, various hypotheses about the pathogenesis of depression have been supported, and people have begun to recognize that depression is the result of the combined effects of multiple factors, including the neuroendocrine, immune system, and gut microbiota. However, each pathogenic mechanism still needs to be further clarified. At present, treatment methods for depression include psychotherapy, pharmacotherapy, electroconvulsive therapy, and other approaches, and it is usually necessary to consider the patient’s specific symptoms, the severity of the condition, and potential drug interactions when choosing the treatment method. For patients with mild to moderate depression, the most common treatment method is pharmacotherapy. While traditional antidepressants (such as TCAs, MAOIs, and SSRIs) remain dominant in clinical treatment, their adverse effects, issues of drug resistance, and significant withdrawal reactions continue to pose significant challenges in clinical application. Over the past decade, global enthusiasm for the development of new antidepressant drugs has continued to grow, with a large number of candidate molecules targeting novel targets emerging, and their clinical trial data demonstrating breakthrough therapeutic potential. Meanwhile, traditional herbal medicines, due to their multi-component synergistic mechanisms of action (such as salidroside regulating the HPA axis and psilocybin/psilocin regulating 5-HT_2A_), are increasingly gaining attention in the field of depression treatment, offering new directions for developing low-side-effect, multi-pathway intervention strategies.

Although traditional medicine has provided various treatment options for depression, we still need to achieve greater breakthroughs in combating this disease. Notably, in-depth research into existing highly active antidepressants has brought many new drugs (e.g., (R, S)-Ketamine and psilocybin). Traditional herbal medicines are increasingly gaining attention due to their unique mechanisms of action and lower side effects. Systematically exploring their active components and mechanisms of action not only holds promise for providing new options for pharmacological treatment of depression but may also reveal entirely new therapeutic targets. Additionally, in-depth analysis of the molecular mechanisms of depression, development of more rigorous clinical trials collectively constitutes the key pathways to advancing depression treatment toward a more scientific and effective direction.

## Figures and Tables

**Table 1:** Overview of antidepressants (classical and emerging agents).

Class	Examples	Primary Mechanism	Clinical Evidence	Key Limitations	References
MAOIs	Phenelzine, Isocarboxazid	MAO inhibition → ↑ monoamines	Established efficacy in atypical depression	Tyramine restrictions, hypertensive risk	[126,127,128,143]
TCAs	Amitriptyline, Imipramine	5-HT/NE reuptake inhibition	Broad efficacy, low cost	Anticholinergic effects, cardiotoxicity	[125,130,131,132]
SSRIs	Fluoxetine, Sertraline	Selective 5-HT reuptake inhibition	First-line for mild-mod MDD	Sexual dysfunction, withdrawal	[133,134,135,136,144]
SNRIs	Venlafaxine, Duloxetine	Dual 5-HT/NE reuptake inhibition	Rapid onset (some agents)	Hypertension, nausea	[137,139,140,141,142]
Atypical	Bupropion, Agomelatine	Varied (e.g., NDRI, melatonergic agonism)	Fewer anticholinergic effects	Sedation, activation	[143,144,145,146,147,148,149]
Herbal Agents	Hypericum perforatum (Hypericin)	SERT/NET inhibition, GABA modulation	Established: Efficacy vs. placebo (mild-mod MDD)	CYP450 interactions	[150,151,152,153]
	Rhodiola rosea (Salidroside)	MAO inhibition, ↓IL-6, ↑BDNF	Probable: Improved HAM-D scores (RCTs)	Limited long-term data	[154,155,156,157]
	Panax ginseng (Ginsenosides)	HPA modulation, ↑BDNF/TrkB	Preclinical: Stress resilience models	No robust human RCTs	[158,159,160,161,162]
Novel Agents	(R,S)-Ketamine	NMDA antagonism → ↑ AMPAR activation	FDA-approved: TRD, acute suicidality	Transient dissociation, abuse potential	[163,164,165,166]
	Psilocybin	5-HT_2A_ agonism → neural plasticity	Phase II: Breakthrough therapy (TRD)	Hallucinogenic effects	[167,168]
	Cariprazine (adjunct)	D_2_/D_3_ partial agonism	FDA-approved: Adjunct for MDD/BD	Akathisia, metabolic effects	[169,170,171]

Clinical Evidence Levels: Established = Meta-analysis support; Probable = ≥2 RCTs; Preclinical = Animal models only. ↑ Increases/Upregulates/Elevates; ↓ Decreases/Downregulates/Suppresses; → Leads to/Results in/Causes.

**Table 2:** Critical appraisal of evidence on depression pathogenesis and treatment (GRADE: High [H], Moderate [M], Low [L], Very Low [VL]; CI: Confidence Interval).

Evidence Domain	Key Limitations	Evidence Quality	Conflicting Findings/Gaps	References
Biomarker Heterogeneity	High inter-study variability in cytokine/oxidative stress levels; lack of standardized assays.	L (Inconsistency ^1^)	IL-6 elevation in depression: 67% studies vs. 33% null (*n* = 45 meta-analyses)	[39,40,41,43,44,45,61,62]
IDO Inhibitor Translation	Compensatory TDO activation in humans; low BBB permeability of candidates; no predictive biomarkers.	VL (Indirectness ^2^)	Preclinical efficacy (rodent despair tests) vs. null human RCTs (*n* = 3)	[42,43,44,45,46,47,48,97]
Microbiome Reproducibility	Geographical/dietary confounders; low agreement on depression-associated taxa (F/B ratio, Bacteroides spp.).	L (Imprecision ^3^)	FMT-induced depressive phenotypes: replicable in rodents (*n* = 5) but not primates	[82,83,84,85,86,87,97,105,106,107]
Neuroplasticity Targets	BDNF as treatment biomarker: inconsistent correlation with symptom improvement (HDRS ∆).	M (Publication bias ^4^)	BDNF ↑ post-SSRI: 71% studies (95% CI: 62–79%); no ∆ in 29%	[70,71,72,73,74,75,79,80,81]

^1^ Inconsistency: Wide prediction intervals in meta-analyses. ^2^ Indirectness: Extrapolation from animal models. ^3^ Imprecision: Small sample sizes; heterogeneous cohorts. ^4^ Publication bias: Positive results overrepresented. ↑ Upregulates.

## Data Availability

Not applicable.
